# New mixed aluminium–chromium diarsenate

**DOI:** 10.1107/S2056989017001797

**Published:** 2017-02-10

**Authors:** Mohamad Alem Bouhassine, Habib Boughzala

**Affiliations:** a Laboratoire de Materiaux et Cristallochimie, Faculté des Sciences de Tunis, Université de Tunis El Manar, 2092 Manar II Tunis, Tunisia

**Keywords:** crystal structure, single crystal diffraction, mixed occupancy, channel structure, diarsenate

## Abstract

Potassium chromium aluminium diarsenate, KCr_1/4_Al_3/4_As_2_O_7_, was prepared by solid-state reaction. The structure consists of (Cr_1/4_/Al_3/4_)O_6_ octa­hedra and As_2_O_7_ diarsenate groups sharing corners to build up a three-dimensional anionic framework. The potassium cations are located in wide channels running along the *c*-axis direction. The crystal structure is isostructural with the triclinic *A* ^I^ *M* ^III^ *X*_2_O_7_ (*A* ^I^ = alkali metal; *M* ^III^ = Al, Cr, Fe; *X* = As, P) compounds. However, the *M* ^III^ octa­hedrally coordinated site is 25% partially occupied by chromium and 75% by aluminium.

## Abstract 2

The title compound structure consists of (Cr_1/4_/Al_3/4_)O_6_ octa­hedra and As_2_O_7_ diarsenate groups sharing corners to build up a three-dimensional anionic framework. The potassium cations are located in wide channels running along the *c*-axis direction.

## Chemical context   

In recent years, inorganic metal phosphates/arsenates with formula *A* ^I^ *M* ^III^ *X*_2_O_7_ (*A* ^I^ = alkali metal; *M* ^III^ = Al, Cr, Fe; *X* = As, P) have been part of intensive research activities, with crystals grown either from high-temperature solid-state reactions or under aqueous solution conditions. The crystal chemistry of these compounds with *X*_2_O_7_ groups reveals a large structural variety accompanied in some cases by inter­esting magnetic, electric, optical, or thermal expansion properties. Focusing on compounds with *M* ^III^ = Cr, it is noticeable that the corresponding diphosphates have been studied extensively, in contrast to the scarcely studied chromium diarsenates. The title structure is isostructural with the *A* ^I^Cr^III^As_2_O_7_ family; nevertheless, in this crystal structure some of the chromium ions are partly substituted by aluminium in an octahedrally coordinated site. Herein, the preparation and crystal structure of KCr_1/4_Al_3/4_As_2_O_7_ is reported. It is one of a series of new potassium chromium–aluminum diarsenate compounds recently isolated by our group.

## Structural commentary   

**Figure 1:**
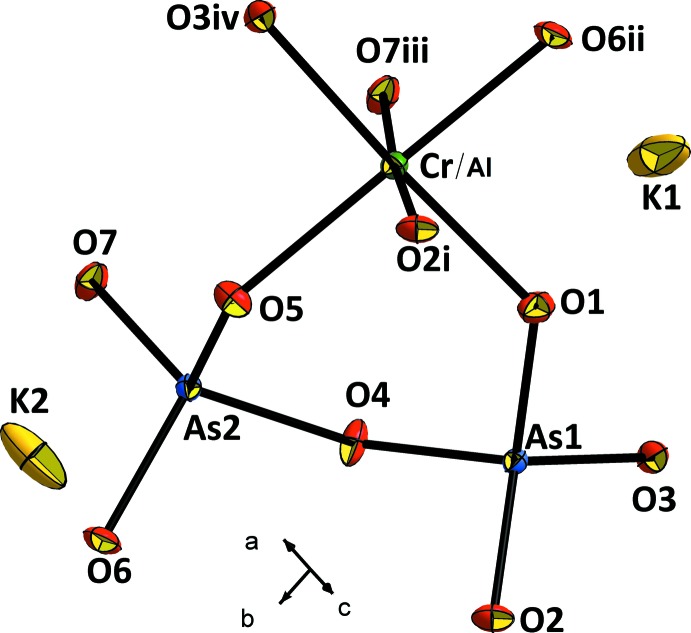
A view of the asymmetric unit of the title compound completed by equivalent atomic positions, showing the principal structural units. Displacement ellipsoids are drawn at the 50% probability level. [Symmetry codes: (i) −*x* + 1, −*y* + 1, −*z* + 1; (ii) *x*, *y* + 1, *z*; (iii) −*x* + 1, −*y* + 1, −*z* + 2; (iv) *x* + 1, *y*, *z*.]

**Figure 2:**
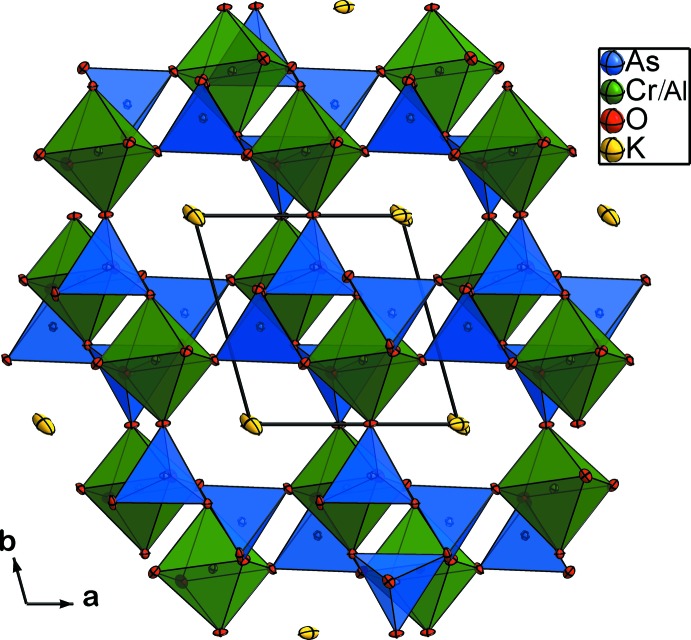
Projection of the KCr_1/4_Al_3/4_As_2_O_7_ structure showing the channels parallel to [001] in which the K^+^ cations are located.

The structure of KCr_1/4_Al_3/4_As_2_O_7_ can be described as a three-dimensional framework of [(Cr/Al)As_2_O_7_]^−^ anions built up from corner-sharing (Cr/Al)O_6_ octa­hedra and As_2_O_7_ groups. The (Cr/Al)O_6_ octa­hedron shares its six corners with five diarsenate groups while the As_2_O_7_ anion shares all of its six corners with five octa­hedra; the inter­connection between the polyhedra results in centrosymmetric (Cr/Al)As_2_O_11_ units (Fig. 1 ▸). The framework can also be described as been formed by polyhedral parallel layers, as in many isoformular compounds, leaving empty channels running along the *c* axis in which the K^+^ cations are located (Fig. 2 ▸).

In this structure, the aluminium Al^III^ and the chromium Cr^III^ cations share the same (2*i*) crystallographic site. These cations are surrounded by oxygen atoms in an octa­hedral coordination with an average bond length (Cr/Al)—O of 1.920 (14) Å. The presence of the Cr^III^ cations is proved by the shortening of the Cr—O bond length compared to Al^III^—O. In fact, according to similar studies (Bouhassine & Boughzala, 2014 ▸, 2015 ▸) the average Al^III^—O and Cr^III^—O bond lengths in octa­hedral coordination are 1.907 and 1.979 Å, respectively.

The two arsenic atoms in the unit cell are tetra­hedrally coordinated. The AsO_4_ polyhedra connected *via* the bridging O4 atom into a diarsenate As_2_O_7_ anion. Like in the related triclinic structures of KAlAs_2_O_7_ (Boughzala & Jouini, 1995 ▸) and RbAlAs_2_O_7_ (Boughzala *et al.*, 1993 ▸), the As—O distances involving the bridging O4 atom are the longest (Table 1 ▸). The As1—O4—As2 bridging angle of 118.50 (14)° in the title structure is similar to that in the reported isotypic structures of CsCrAs_2_O_7_ [118.7 (2)°; Bouhassine & Boughzala, 2015 ▸] and KAlAs_2_O_7_ [118.3 (2)°; Boughzala & Jouini, 1995 ▸]. The O—As—O bond angles for As1 and As2 span the ranges 103.99 (12) to 117.41 (13) and 106.34 (13) to 113.63 (12), respectively, reflecting a slight distortion of each AsO_4_ tetra­hedron.

**Figure 3:**
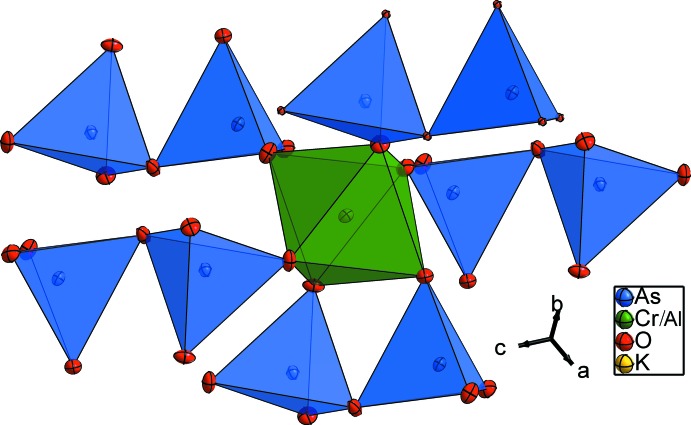
The environment of the (Cr/Al)O_6_ octa­hedron in the structure of KCr_1/4_Al_3/4_As_2_O_7_.

**Figure 4:**
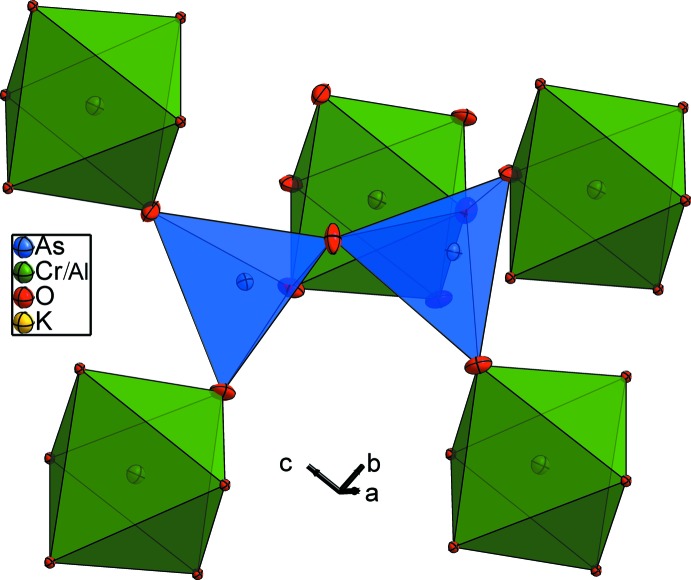
The environment of the diarsenate group As_2_O_7_ in the title structure.

The (Cr/Al) cations are in a slightly distorted octa­hedral oxygen coordination with (Cr/Al)—O distances ranging from 1.898 (3) to 1.940 (3) Å, and with O—(Cr/Al)—O angles ranging from 85.28 (11) to 92.23 (12)° and from 177.25 (11) to 176.41 (11)°. Each (Cr/Al)O_6_ octa­hedron is linked by its six vertices to five As_2_O_7_ anions. Two corners are joined to the same diarsenate group (Fig. 3 ▸). On the other hand, each As_2_O_7_ anion is surrounded by five (Cr/Al)O_6_ units (Fig. 4 ▸).

**Figure 5:**
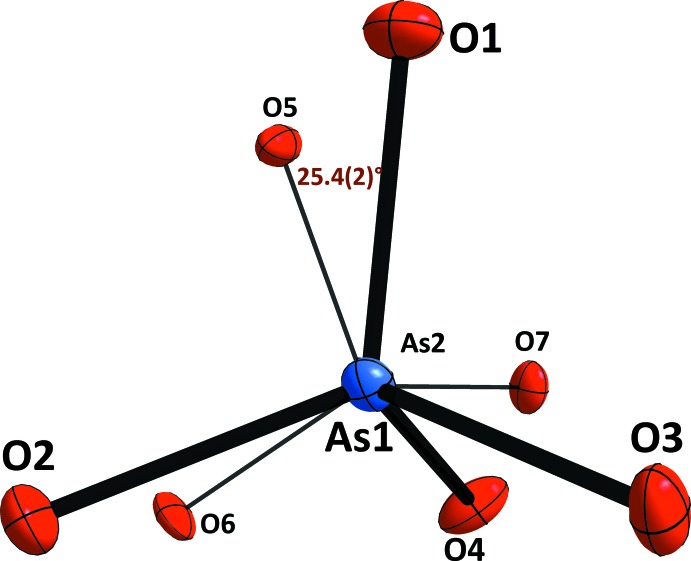
View parallel to the As1—As2 direction, emphasizing the nearly eclipsed conformation of the diarsenate anion.

It is worth mentioning that members of the related alumin­ium diarsenate family *A* ^I^AlAs_2_O_7_ (*A* ^I^ = K, Rb, Tl, Cs; Boughzala & Jouini, 1992 ▸) crystallize in the triclinic space group *P* 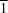 and are classified as type II (Durif & Averbuch-Pouchot, 1996 ▸); the diarsenate groups have a different conformational orientation compared to that of the title structure, which belongs to the type I family of *A* ^I^ *M* ^III^ *X*_2_O_7_ diarsenates. In fact, the diarsenate tetra­hedra are in a nearly eclipsed conformation with an O1—As1—As2—O5 torsion angle of 25.4 (2)°, as shown in Fig. 5 ▸. The corresponding angle is 158.8 (2)° for KAlAs_2_O_7_ (Boughzala & Jouini, 1995 ▸).

**Figure 6:**
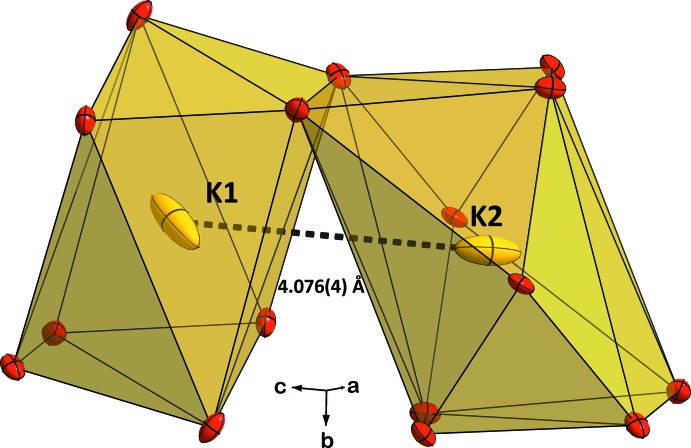
The eight- and ten-coordinated K1 and K2 atoms (polyhedral plot) in the structure of KCr_1/4_Al_3/4_As_2_O_7_.

The potassium cations lodge in two independent special positions in the unit cell, located in wide channels that are delimited by the anionic framework and run along the *c*-axis direction. The K1 and K2 cations are surrounded by eight and ten oxygen atoms, respectively (Fig. 6 ▸), with K—O distances ranging from 2.769 (3) to 3.246 (3) Å and from 2.806 (3) to 3.205 (3) Å, respectively, forming irregular coordination polyhedra, as often occurs with this cation in homologous structures.

## Database survey   

The structure of KAlP_2_O_7_ (Ng & Calvo, 1973 ▸) was the first to be published for the *A* ^I^ *M* ^III^ *X*_2_O_7_ family (*X* = As, P). Afterwards, based on different substitutions and combinations, a large number of different phases were isolated and characterized crystallographically. Replacement of the cations can improve the structural and physical properties, but also affects the coordination numbers, the distortion of the coordination polyhedra and the conformation of the *X*_2_O_7_ groups. In addition, the crystal symmetry can be affected. The structures are triclinic, in space group *P* 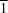 with two formulas units, for the diarsenate compounds *A* ^I^AlAs_2_O_7_ (*A* ^I^ = K, Rb, Tl, Cs) (Boughzala & Jouini, 1992 ▸; Boughzala *et al.*, 1993 ▸; Boughzala & Jouini; 1995 ▸), whereas the diphosphates are generally monoclinic. The isotypic *A* ^I^CrP_2_O_7_ phases crystallize in space group *P*2_1_/*c* for *A* ^I^ = Na (Bohatý *et al.*, 1982 ▸), K (Gentil *et al.*, 1997 ▸), Rb (Zhao & Li, 2011 ▸) and Cs (Linde & Gorbunova, 1982 ▸). The same applies for the *A* ^I^FeP_2_O_7_ phases for *A* ^I^ = Na (Gabelica-Robert *et al.*, 1982 ▸) and K (Riou *et al.*, 1988 ▸). However, the two Li-containing phases Li*M*P_2_O_7_ show a symmetry reduction to space group *P*2_1_ (*M* = Cr, Ivashkevich *et al.*, 2007 ▸; *M* = Fe, Riou *et al.*, 1990 ▸). CsCrAs_2_O_7_ (Bouhassine & Boughzala, 2015 ▸) is the first phase of the *A* ^I^CrAs_2_O_7_ family to crystallize in the *P*2_1_/c space group.

## Synthesis and crystallization   

The crystals of the title compound were obtained from heating a mixture of KNO_3_, Cr_2_O_3_ and NH_4_H_2_AsO_4_, with a K:Cr:As molar ratio of 2:1:2. In order to eliminate volatile products, the sample was placed in a porcelain crucible and slowly heated under atmospheric conditions to 673 K and kept for 12 h. In a second step, the crucible was progressively heated at 1123 K for 10 days and then slowly cooled down at a rate of 5 K/24h to 923 K and finally allowed to cool radiatively to room temperature. A long wash with boiling water liberated green crystals. Manifestly, the aluminium present in the studied composition is coming from the porcelain crucible.

## Refinement   

**Figure 7:**
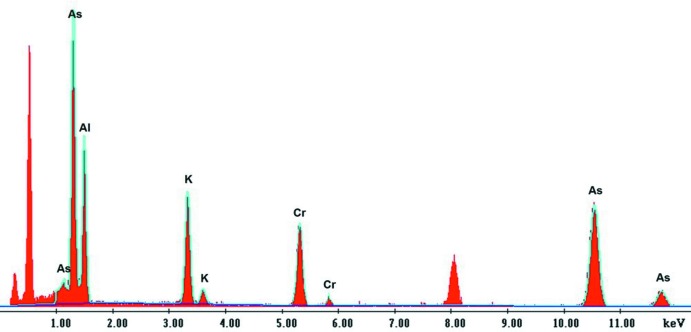
Qualitative elemental composition determined by TEM (Fei Tecnai G20 STEM microscope). (Low-energy unlabelled peaks are related to oxygen and the one around 8 keV is attributed to the copper sample holder).

Crystal data, data collection and structure refinement details are summarized in Table 2 ▸. The 2*i* site was initially refined as being entirely occupied by chromium ions with reliability factor *R*(*F* ^2^) = 0.053. Trying to improve the convergence factor, the occupation rate of the 2*i* site was refined, leading to *R*(*F* ^2^) = 0.023 and a partial occupancy of 67%. Occupied by just Cr^III^, this occupancy is insufficient to achieve electric neutrality in the empirical formula. To ensure the electroneutrality, many propositions were considered such as the existence of some vacancies in the positions of the oxygen atoms, or the contribution of more than one oxidation state of chromium in the 2*i* site. The most reasonable idea was to consider a competitive presence of Cr^III^ and Al^III^ in the same crystallographic site endowed with the same *U*_*ij*_ parameters. The aluminium has obviously diffused from the porcelain crucible. The last refinement steps lead to the final formula KCr_1/4_Al_3/4_As_2_O_7._ The presence of both aluminium and chromium in the structure was confirmed by TEM as shown in Fig. 7 ▸.

## Supplementary Material

Crystal structure: contains datablock(s) I. DOI: 10.1107/S2056989017001797/pj2041sup1.cif

Structure factors: contains datablock(s) I. DOI: 10.1107/S2056989017001797/pj2041Isup2.hkl

CCDC reference: 1530620

Additional supporting information:  crystallographic information; 3D view; checkCIF report

## Figures and Tables

**Table 1:** Selected geometric parameters (Å, °)

As1—O2	1.659 (2)	As2—O4	1.761 (3)
As1—O3^i^	1.669 (3)	(Cr/Al)—O6^ii^	1.898 (3)
As1—O1	1.669 (3)	(Cr/Al)—O3	1.915 (3)
As1—O4	1.776 (2)	(Cr/Al)—O1	1.919 (3)
As2—O6	1.654 (2)	(Cr/Al)—O5	1.925 (3)
As2—O7	1.663 (2)	(Cr/Al)—O2^iii^	1.925 (3)
As2—O5	1.675 (2)	(Cr/Al)—O7^iv^	1.940 (3)
			
As2—O4—As1	118.50 (14)		

Symmetry codes: (i) 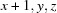; (ii) 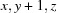; (iii) 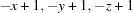; (iv) 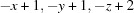.

**Table 2:** Experimental details

Crystal data	
Chemical formula	KCr_0.25_Al_0.75_As_2_O_7_
*M*_r_	334.17
Crystal system, space group	Triclinic, *P* 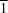
Temperature (K)	293
*a*, *b*, *c* (Å)	6.243 (3), 6.349 (3), 8.153 (4)
α, β, γ (°)	96.57 (2), 104.45 (3), 103.08 (4)
*V* (Å^3^)	299.8 (8)
*Z*	2
Radiation type	Mo *K*α
μ (mm^−1^)	12.37
Crystal size (mm)	0.40 × 0.30 × 0.20

Data collection	
Diffractometer	Enraf–Nonius CAD-4
Absorption correction	ψ scan (North *et al.*, 1968 ▸)
*T*_min_, *T*_max_	0.079, 0.182
No. of measured, independent and observed [*I* > 2σ(*I*)] reflections	1628, 1479, 1306
*R*_int_	0.014
(sin θ/λ)_max_ (Å^−1^)	0.702

Refinement	
*R*[*F* ^2^ > 2σ(*F* ^2^)], *wR*(*F* ^2^), *S*	0.023, 0.059, 1.08
No. of reflections	1479
No. of parameters	104
Δρ_max_, Δρ_min_ (e Å^−3^)	0.59, −1.09

Computer programs: *CAD-4 EXPRESS* (Enraf–Nonius, 1994 ▸), *XCAD4* (Harms & Wocadlo, 1995 ▸), *SHELXS97* (Sheldrick, 2008 ▸), *SHELXL97* (Sheldrick, 2008 ▸), *DIAMOND* (Brandenburg, 2008 ▸) and *publCIF* (Westrip, 2010 ▸).

## supplementary crystallographic information

### Crystal data

**Table:**

KCr_0.25_Al_0.75_As_2_O_7_	*Z* = 2
*M**_r_* = 334.17	*F*(000) = 314
Triclinic, *P*1	*D*_x_ = 3.702 Mg m^−^^3^
Hall symbol: -p 1	Mo *K*α radiation, λ = 0.71073 Å
*a* = 6.243 (3) Å	Cell parameters from 25 reflections
*b* = 6.349 (3) Å	θ = 3.8–27°
*c* = 8.153 (4) Å	µ = 12.37 mm^−^^1^
α = 96.57 (2)°	*T* = 293 K
β = 104.45 (3)°	Triclinic, green
γ = 103.08 (4)°	0.40 × 0.30 × 0.20 mm
*V* = 299.8 (8) Å^3^	

### Data collection

**Table:**

Enraf–Nonius CAD-4 diffractometer	1306 reflections with *I* > 2σ(*I*)
Radiation source: fine-focus sealed tube	*R*_int_ = 0.014
Graphite monochromator	θ_max_ = 30.0°, θ_min_ = 2.6°
ω/2θ scans	*h* = 0→7
Absorption correction: ψ scan (North *et al.*, 1968)	*k* = −8→8
*T*_min_ = 0.079, *T*_max_ = 0.182	*l* = −11→11
1628 measured reflections	2 standard reflections every 120 min
1479 independent reflections	intensity decay: 1.1%

### Refinement

**Table:**

Refinement on *F*^2^	0 restraints
Least-squares matrix: full	Primary atom site location: structure-invariant direct methods
*R*[*F*^2^ > 2σ(*F*^2^)] = 0.023	Secondary atom site location: difference Fourier map
*wR*(*F*^2^) = 0.059	*w* = 1/[σ^2^(*F*_o_^2^) + (0.0303*P*)^2^ + 0.5924*P*] where *P* = (*F*_o_^2^ + 2*F*_c_^2^)/3
*S* = 1.08	(Δ/σ)_max_ < 0.001
1479 reflections	Δρ_max_ = 0.59 e Å^−^^3^
104 parameters	Δρ_min_ = −1.09 e Å^−^^3^

### Special details

**Table:**

Geometry. All esds (except the esd in the dihedral angle between two l.s. planes) are estimated using the full covariance matrix. The cell esds are taken into account individually in the estimation of esds in distances, angles and torsion angles; correlations between esds in cell parameters are only used when they are defined by crystal symmetry. An approximate (isotropic) treatment of cell esds is used for estimating esds involving l.s. planes.
Refinement. Refinement of F^2^ against ALL reflections. The weighted R-factor wR and goodness of fit S are based on F^2^, conventional R-factors R are based on F, with F set to zero for negative F^2^. The threshold expression of F^2^ > 2sigma(F^2^) is used only for calculating R-factors(gt) etc. and is not relevant to the choice of reflections for refinement. R-factors based on F^2^ are statistically about twice as large as those based on F, and R- factors based on ALL data will be even larger.

### Fractional atomic coordinates and isotropic or equivalent isotropic displacement parameters (Å^2^)

**Table:**

	*x*	*y*	*z*	*U*_iso_*/*U*_eq_	Occ. (<1)
As1	0.82649 (6)	0.53279 (5)	0.68995 (4)	0.00590 (10)	
As2	0.48557 (6)	0.24607 (5)	0.83512 (4)	0.00587 (10)	
Cr	0.37535 (14)	0.69197 (13)	0.72579 (10)	0.0054 (3)	0.255 (6)
Al	0.37535 (14)	0.69197 (13)	0.72579 (10)	0.0054 (3)	0.745 (6)
O1	0.6626 (4)	0.7081 (4)	0.6757 (3)	0.0109 (5)	
O2	0.7856 (4)	0.3500 (4)	0.5149 (3)	0.0105 (5)	
O3	0.0934 (4)	0.6909 (4)	0.7793 (3)	0.0097 (5)	
O4	0.7722 (4)	0.3747 (4)	0.8485 (3)	0.0108 (5)	
O5	0.3164 (4)	0.3800 (4)	0.7206 (3)	0.0100 (5)	
O6	0.4234 (5)	−0.0032 (4)	0.7201 (3)	0.0113 (5)	
O7	0.4552 (4)	0.2466 (4)	1.0321 (3)	0.0106 (5)	
K1	1.0000	1.0000	1.0000	0.0484 (5)	
K2	0.0000	0.0000	0.5000	0.0364 (4)	

### Atomic displacement parameters (Å^2^)

**Table:**

	*U*^11^	*U*^22^	*U*^33^	*U*^12^	*U*^13^	*U*^23^
As1	0.00519 (19)	0.00627 (16)	0.00641 (15)	0.00121 (12)	0.00247 (12)	0.00063 (11)
As2	0.00655 (19)	0.00535 (15)	0.00580 (15)	0.00124 (12)	0.00216 (12)	0.00115 (10)
Cr	0.0047 (5)	0.0053 (4)	0.0062 (4)	0.0015 (3)	0.0019 (3)	0.0005 (3)
Al	0.0047 (5)	0.0053 (4)	0.0062 (4)	0.0015 (3)	0.0019 (3)	0.0005 (3)
O1	0.0127 (13)	0.0117 (11)	0.0122 (11)	0.0066 (10)	0.0060 (10)	0.0049 (9)
O2	0.0122 (13)	0.0107 (11)	0.0079 (10)	0.0041 (10)	0.0022 (9)	−0.0015 (8)
O3	0.0070 (12)	0.0095 (11)	0.0097 (10)	−0.0010 (9)	0.0023 (9)	−0.0026 (8)
O4	0.0056 (12)	0.0159 (12)	0.0119 (11)	0.0017 (10)	0.0026 (9)	0.0079 (9)
O5	0.0089 (13)	0.0076 (10)	0.0113 (11)	0.0025 (9)	−0.0010 (9)	0.0011 (8)
O6	0.0163 (14)	0.0064 (11)	0.0109 (11)	0.0033 (10)	0.0045 (10)	−0.0016 (8)
O7	0.0106 (13)	0.0139 (11)	0.0069 (10)	0.0011 (10)	0.0038 (9)	0.0012 (9)
K1	0.0302 (9)	0.0408 (9)	0.0615 (11)	−0.0093 (7)	0.0253 (8)	−0.0310 (8)
K2	0.0354 (9)	0.0148 (6)	0.0393 (8)	0.0055 (6)	−0.0209 (6)	−0.0002 (5)

### Geometric parameters (Å, º)

**Table:**

As1—O2	1.659 (2)	O3—K2^v^	3.202 (3)
As1—O3^i^	1.669 (3)	O4—K1^iv^	3.246 (3)
As1—O1	1.669 (3)	O5—K2	2.834 (3)
As1—O4	1.776 (2)	O6—Al^iv^	1.898 (3)
As1—K1	3.4388 (16)	O6—Cr^iv^	1.898 (3)
As1—K2^ii^	3.5783 (15)	O6—K2	2.806 (3)
As2—O6	1.654 (2)	O7—Al^vii^	1.940 (3)
As2—O7	1.663 (2)	O7—Cr^vii^	1.940 (3)
As2—O5	1.675 (2)	O7—K1^iii^	2.849 (3)
As2—O4	1.761 (3)	K1—O3^i^	2.769 (3)
As2—K2	3.441 (2)	K1—O3^ix^	2.769 (3)
As2—K1^iii^	3.7159 (17)	K1—O7^vii^	2.849 (3)
As2—K1^iv^	3.8935 (18)	K1—O7^ii^	2.849 (3)
Cr—O6^v^	1.898 (3)	K1—O1^x^	3.032 (3)
Cr—O3	1.915 (3)	K1—O4^xi^	3.246 (3)
Cr—O1	1.919 (3)	K1—O4^v^	3.246 (3)
Cr—O5	1.925 (3)	K1—As1^x^	3.4388 (16)
Cr—O2^vi^	1.925 (3)	K1—As2^ii^	3.7160 (17)
Cr—O7^vii^	1.940 (3)	K1—As2^vii^	3.7160 (17)
Cr—K2^v^	3.6674 (17)	K2—O6^xii^	2.806 (3)
Cr—K1	3.902 (2)	K2—O5^xii^	2.834 (3)
O1—K1	3.032 (3)	K2—O2^xiii^	2.848 (3)
O1—K2^ii^	3.205 (3)	K2—O2^viii^	2.848 (3)
O2—Al^vi^	1.925 (3)	K2—O3^iv^	3.202 (3)
O2—Cr^vi^	1.925 (3)	K2—O3^xiv^	3.202 (3)
O2—K2^i^	2.848 (3)	K2—O1^vi^	3.205 (3)
O3—As1^viii^	1.669 (3)	K2—O1^iii^	3.205 (3)
O3—K1^viii^	2.769 (3)	K2—As2^xii^	3.441 (2)
			
O2—As1—O3^i^	116.44 (13)	O3^ix^—K1—O4^xi^	119.81 (7)
O2—As1—O1	117.41 (13)	O7^vii^—K1—O4^xi^	93.40 (8)
O3^i^—As1—O1	104.67 (13)	O7^ii^—K1—O4^xi^	86.60 (8)
O2—As1—O4	105.16 (13)	O1^x^—K1—O4^xi^	82.64 (7)
O3^i^—As1—O4	103.99 (12)	O1—K1—O4^xi^	97.36 (7)
O1—As1—O4	108.17 (12)	O3^i^—K1—O4^v^	119.81 (7)
O2—As1—K1	164.68 (9)	O3^ix^—K1—O4^v^	60.19 (7)
O3^i^—As1—K1	52.76 (9)	O7^vii^—K1—O4^v^	86.60 (8)
O1—As1—K1	61.84 (9)	O7^ii^—K1—O4^v^	93.40 (8)
O4—As1—K1	88.94 (9)	O1^x^—K1—O4^v^	97.36 (7)
O2—As1—K2^ii^	94.71 (9)	O1—K1—O4^v^	82.64 (7)
O3^i^—As1—K2^ii^	63.46 (9)	O4^xi^—K1—O4^v^	180.000 (1)
O1—As1—K2^ii^	63.56 (9)	O3^i^—K1—As1^x^	151.32 (5)
O4—As1—K2^ii^	159.92 (8)	O3^ix^—K1—As1^x^	28.68 (6)
K1—As1—K2^ii^	71.00 (4)	O7^vii^—K1—As1^x^	112.10 (6)
O6—As2—O7	113.63 (12)	O7^ii^—K1—As1^x^	67.90 (6)
O6—As2—O5	106.34 (13)	O1^x^—K1—As1^x^	29.03 (5)
O7—As2—O5	111.83 (13)	O1—K1—As1^x^	150.97 (5)
O6—As2—O4	106.77 (13)	O4^xi^—K1—As1^x^	109.51 (6)
O7—As2—O4	109.74 (12)	O4^v^—K1—As1^x^	70.49 (6)
O5—As2—O4	108.26 (12)	O3^i^—K1—As1	28.68 (5)
O6—As2—K2	53.92 (10)	O3^ix^—K1—As1	151.32 (6)
O7—As2—K2	116.01 (10)	O7^vii^—K1—As1	67.90 (6)
O5—As2—K2	54.99 (9)	O7^ii^—K1—As1	112.10 (6)
O4—As2—K2	134.24 (8)	O1^x^—K1—As1	150.97 (5)
O6—As2—K1^iii^	83.81 (10)	O1—K1—As1	29.03 (5)
O7—As2—K1^iii^	46.83 (9)	O4^xi^—K1—As1	70.49 (6)
O5—As2—K1^iii^	88.35 (10)	O4^v^—K1—As1	109.51 (6)
O4—As2—K1^iii^	156.13 (8)	As1^x^—K1—As1	180.0
K2—As2—K1^iii^	69.32 (4)	O3^i^—K1—As2^ii^	68.09 (6)
O6—As2—K1^iv^	72.50 (10)	O3^ix^—K1—As2^ii^	111.91 (6)
O7—As2—K1^iv^	85.22 (9)	O7^vii^—K1—As2^ii^	154.81 (5)
O5—As2—K1^iv^	160.88 (9)	O7^ii^—K1—As2^ii^	25.19 (5)
O4—As2—K1^iv^	55.72 (8)	O1^x^—K1—As2^ii^	78.58 (6)
K2—As2—K1^iv^	126.37 (3)	O1—K1—As2^ii^	101.42 (6)
K1^iii^—As2—K1^iv^	110.23 (3)	O4^xi^—K1—As2^ii^	94.66 (6)
O6^v^—Cr—O3	89.05 (12)	O4^v^—K1—As2^ii^	85.34 (6)
O6^v^—Cr—O1	88.35 (12)	As1^x^—K1—As2^ii^	87.50 (4)
O3—Cr—O1	177.25 (11)	As1—K1—As2^ii^	92.50 (4)
O6^v^—Cr—O5	177.24 (11)	O3^i^—K1—As2^vii^	111.91 (6)
O3—Cr—O5	90.41 (12)	O3^ix^—K1—As2^vii^	68.09 (6)
O1—Cr—O5	92.23 (12)	O7^vii^—K1—As2^vii^	25.19 (5)
O6^v^—Cr—O2^vi^	85.28 (11)	O7^ii^—K1—As2^vii^	154.81 (5)
O3—Cr—O2^vi^	88.97 (12)	O1^x^—K1—As2^vii^	101.42 (6)
O1—Cr—O2^vi^	91.72 (12)	O1—K1—As2^vii^	78.58 (6)
O5—Cr—O2^vi^	92.00 (11)	O4^xi^—K1—As2^vii^	85.34 (6)
O6^v^—Cr—O7^vii^	91.24 (11)	O4^v^—K1—As2^vii^	94.66 (6)
O3—Cr—O7^vii^	91.73 (11)	As1^x^—K1—As2^vii^	92.50 (4)
O1—Cr—O7^vii^	87.42 (12)	As1—K1—As2^vii^	87.50 (4)
O5—Cr—O7^vii^	91.49 (11)	As2^ii^—K1—As2^vii^	180.0
O2^vi^—Cr—O7^vii^	176.44 (11)	O6—K2—O6^xii^	180.0
O6^v^—Cr—K2^v^	48.75 (9)	O6—K2—O5	56.38 (8)
O3—Cr—K2^v^	60.75 (8)	O6^xii^—K2—O5	123.62 (8)
O1—Cr—K2^v^	117.88 (8)	O6—K2—O5^xii^	123.62 (8)
O5—Cr—K2^v^	128.80 (9)	O6^xii^—K2—O5^xii^	56.38 (8)
O2^vi^—Cr—K2^v^	50.16 (8)	O5—K2—O5^xii^	180.0
O7^vii^—Cr—K2^v^	127.49 (8)	O6—K2—O2^xiii^	54.52 (8)
O6^v^—Cr—K1	71.06 (9)	O6^xii^—K2—O2^xiii^	125.48 (8)
O3—Cr—K1	128.54 (8)	O5—K2—O2^xiii^	109.72 (8)
O1—Cr—K1	49.56 (8)	O5^xii^—K2—O2^xiii^	70.28 (8)
O5—Cr—K1	111.31 (9)	O6—K2—O2^viii^	125.48 (8)
O2^vi^—Cr—K1	133.48 (9)	O6^xii^—K2—O2^viii^	54.52 (8)
O7^vii^—Cr—K1	44.10 (8)	O5—K2—O2^viii^	70.28 (8)
K2^v^—Cr—K1	119.78 (4)	O5^xii^—K2—O2^viii^	109.72 (8)
As1—O1—Cr	130.98 (15)	O2^xiii^—K2—O2^viii^	180.0
As1—O1—K1	89.13 (11)	O6—K2—O3^iv^	52.34 (7)
Cr—O1—K1	101.64 (10)	O6^xii^—K2—O3^iv^	127.66 (7)
As1—O1—K2^ii^	88.65 (10)	O5—K2—O3^iv^	93.39 (7)
Cr—O1—K2^ii^	139.96 (11)	O5^xii^—K2—O3^iv^	86.61 (7)
K1—O1—K2^ii^	81.57 (8)	O2^xiii^—K2—O3^iv^	52.42 (7)
As1—O2—Al^vi^	136.04 (15)	O2^viii^—K2—O3^iv^	127.58 (7)
As1—O2—Cr^vi^	136.04 (15)	O6—K2—O3^xiv^	127.66 (7)
Al^vi^—O2—Cr^vi^	0.00 (8)	O6^xii^—K2—O3^xiv^	52.34 (7)
As1—O2—K2^i^	125.20 (12)	O5—K2—O3^xiv^	86.61 (8)
Al^vi^—O2—K2^i^	98.57 (10)	O5^xii^—K2—O3^xiv^	93.39 (7)
Cr^vi^—O2—K2^i^	98.57 (10)	O2^xiii^—K2—O3^xiv^	127.58 (7)
As1^viii^—O3—Cr	131.84 (14)	O2^viii^—K2—O3^xiv^	52.42 (7)
As1^viii^—O3—K1^viii^	98.56 (11)	O3^iv^—K2—O3^xiv^	180.0
Cr—O3—K1^viii^	129.00 (12)	O6—K2—O1^vi^	80.54 (8)
As1^viii^—O3—K2^v^	88.74 (10)	O6^xii^—K2—O1^vi^	99.46 (8)
Cr—O3—K2^v^	87.81 (9)	O5—K2—O1^vi^	64.92 (8)
K1^viii^—O3—K2^v^	85.79 (7)	O5^xii^—K2—O1^vi^	115.08 (8)
As2—O4—As1	118.50 (14)	O2^xiii^—K2—O1^vi^	92.66 (7)
As2—O4—K1^iv^	97.65 (10)	O2^viii^—K2—O1^vi^	87.34 (7)
As1—O4—K1^iv^	131.00 (12)	O3^iv^—K2—O1^vi^	131.29 (7)
As2—O5—Cr	127.51 (15)	O3^xiv^—K2—O1^vi^	48.71 (7)
As2—O5—K2	96.06 (11)	O6—K2—O1^iii^	99.46 (8)
Cr—O5—K2	135.48 (11)	O6^xii^—K2—O1^iii^	80.54 (8)
As2—O6—Al^iv^	145.71 (15)	O5—K2—O1^iii^	115.08 (8)
As2—O6—Cr^iv^	145.71 (15)	O5^xii^—K2—O1^iii^	64.92 (8)
Al^iv^—O6—Cr^iv^	0.00 (5)	O2^xiii^—K2—O1^iii^	87.34 (7)
As2—O6—K2	97.63 (11)	O2^viii^—K2—O1^iii^	92.66 (7)
Al^iv^—O6—K2	100.69 (11)	O3^iv^—K2—O1^iii^	48.71 (7)
Cr^iv^—O6—K2	100.69 (11)	O3^xiv^—K2—O1^iii^	131.29 (7)
As2—O7—Al^vii^	143.23 (16)	O1^vi^—K2—O1^iii^	180.00 (7)
As2—O7—Cr^vii^	143.23 (16)	O6—K2—As2^xii^	151.55 (5)
Al^vii^—O7—Cr^vii^	0.00 (8)	O6^xii^—K2—As2^xii^	28.45 (5)
As2—O7—K1^iii^	107.98 (11)	O5—K2—As2^xii^	151.05 (5)
Al^vii^—O7—K1^iii^	107.62 (10)	O5^xii^—K2—As2^xii^	28.95 (5)
Cr^vii^—O7—K1^iii^	107.62 (10)	O2^xiii^—K2—As2^xii^	97.04 (6)
O3^i^—K1—O3^ix^	180.000 (1)	O2^viii^—K2—As2^xii^	82.96 (6)
O3^i^—K1—O7^vii^	95.75 (8)	O3^iv^—K2—As2^xii^	112.12 (6)
O3^ix^—K1—O7^vii^	84.25 (8)	O3^xiv^—K2—As2^xii^	67.88 (6)
O3^i^—K1—O7^ii^	84.25 (8)	O1^vi^—K2—As2^xii^	104.03 (6)
O3^ix^—K1—O7^ii^	95.75 (8)	O1^iii^—K2—As2^xii^	75.97 (6)
O7^vii^—K1—O7^ii^	180.0	O6—K2—As2	28.45 (5)
O3^i^—K1—O1^x^	126.04 (8)	O6^xii^—K2—As2	151.55 (5)
O3^ix^—K1—O1^x^	53.96 (8)	O5—K2—As2	28.95 (5)
O7^vii^—K1—O1^x^	126.18 (8)	O5^xii^—K2—As2	151.05 (5)
O7^ii^—K1—O1^x^	53.82 (8)	O2^xiii^—K2—As2	82.96 (6)
O3^i^—K1—O1	53.96 (8)	O2^viii^—K2—As2	97.04 (6)
O3^ix^—K1—O1	126.04 (8)	O3^iv^—K2—As2	67.88 (6)
O7^vii^—K1—O1	53.82 (8)	O3^xiv^—K2—As2	112.12 (6)
O7^ii^—K1—O1	126.18 (8)	O1^vi^—K2—As2	75.97 (6)
O1^x^—K1—O1	180.0	O1^iii^—K2—As2	104.03 (6)
O3^i^—K1—O4^xi^	60.19 (7)	As2^xii^—K2—As2	180.0

Symmetry codes: (i) *x*+1, *y*, *z*; (ii) *x*+1, *y*+1, *z*; (iii) *x*−1, *y*−1, *z*; (iv) *x*, *y*−1, *z*; (v) *x*, *y*+1, *z*; (vi) −*x*+1, −*y*+1, −*z*+1; (vii) −*x*+1, −*y*+1, −*z*+2; (viii) *x*−1, *y*, *z*; (ix) −*x*+1, −*y*+2, −*z*+2; (x) −*x*+2, −*y*+2, −*z*+2; (xi) −*x*+2, −*y*+1, −*z*+2; (xii) −*x*, −*y*, −*z*+1; (xiii) −*x*+1, −*y*, −*z*+1; (xiv) −*x*, −*y*+1, −*z*+1.
